# Evaluation of Xa inhibitors as potential inhibitors of the SARS-CoV-2 Mpro protease

**DOI:** 10.1371/journal.pone.0262482

**Published:** 2022-01-11

**Authors:** Katarzyna Papaj, Patrycja Spychalska, Patryk Kapica, André Fischer, Jakub Nowak, Maria Bzówka, Manuel Sellner, Markus A. Lill, Martin Smieško, Artur Góra

**Affiliations:** 1 Tunneling Group, Biotechnology Centre, Silesian University of Technology, Gliwice, Poland; 2 Department of Pharmaceutical Sciences, Computational Pharmacy, University of Basel, Basel, Switzerland; 3 Faculty of Biochemistry, Biophysics and Biotechnology, Department of Physical Biochemistry, Jagiellonian University, Krakow, Poland; Management and Science University, MALAYSIA

## Abstract

Based on previous large-scale *in silico* screening several factor Xa inhibitors were proposed to potentially inhibit SARS-CoV-2 M^pro^. In addition to their known anticoagulants activity this potential inhibition could have an additional therapeutic effect on patients with COVID-19 disease. In this study we examined the binding of the Apixaban, Betrixaban and Rivaroxaban to the SARS-CoV-2 M^pro^ with the use of the MicroScale Thermophoresis technique. Our results indicate that the experimentally measured binding affinity is weak and the therapeutic effect due to the SARS-CoV-2 M^pro^ inhibition is rather negligible.

## Introduction

In December 2019, a novel virus termed severe acute respiratory syndrome coronavirus 2 (SARS-CoV-2) spread rapidly around the world causing coronavirus disease 2019 (COVID-19). As of October 2021, the number of confirmed infections has reached nearly 250 million causing over four million fatalities [1]. Infections with COVID-19 have been linked to coagulopathies that increase the mortality of patients. In particular, venous thromboembolism (VTE), and sepsis-induced coagulopathy (SIC) ultimately progressing to life-threatening disseminated intravascular coagulation (DIC) were diagnosed in hospitalized patients [2]. The latter is suspected to be associated with inflammation provoking systemic coagulation in a process described as thromboinflammation, and is likely not caused by the inherent nature of the virus. In a recent review, it was recommended to prophylactically treat VTE with anticoagulants in confirmed or suspected COVID-19 patients upon hospital admission. Further, treatment algorithms of many clinical institutions include heparin as well as factor Xa inhibitors to treat COVID-19 patients [3]. As have been reported, the mortality of critically ill COVID-19 patients is reduced if anticoagulants are appropriately applied according to their clinical status. Specifically, patients with high levels of D-dimer and those meeting clinical SIC criteria profit from the administration of anticoagulants. In addition to its role in coagulation, factor Xa has been proposed to increase viral infectivity by cleaving the spike protein of the SARS-CoV virus into its active components, suggesting factor Xa inhibition as a beneficial therapeutic measure for COVID-19 [3–5].

Apixaban, Betrixaban, and Rivaroxaban competitively and directly inhibit factor Xa. This prevents the conversion from prothrombin to thrombin, thereby disrupting the coagulation cascade [6,7]. Such direct factor Xa inhibitors are clinically used to treat or prevent venous thromboembolisms. In addition, antiviral effects due to anticoagulant activity have been proposed for direct factor Xa inhibitors [8–10].

Direct pharmacological intervention to curb viral replication frequently involves targeting essential viral proteases, as evidenced in the treatment of other viral infections such as human immunodeficiency virus (HIV) and hepatitis C virus (HCV) [11,12]. As it was underlined in many studies, the main protease of SARS-CoV-2 (M^pro^) remains the leading molecular target for therapeutics development against the COVID-19 disease, since it is thought to be essential for the viral life cycle [13–15]. Recent screening studies discovered multiple covalent inhibitors of the SARS-CoV-2 M^pro^ and subsequently proved their antiviral efficacy in cellular assays [16,17]. This concludes that the inhibition of the SARS-CoV-2 M^pro^ offers a promising strategy to treat COVID-19.

Strategies for finding potential inhibitors towards SARS-CoV-2 M^pro^ often include performing a massive virtual screening of various libraries [18–22]. Based on virtual screening of a library containing over 600 million compounds, several factor Xa inhibitors that could also potentially inhibit the SARS-CoV-2 M^pro^, were identified [18]. Since the inhibition of factor Xa seems to have favorable effects on the outcome of SARS-CoV-2 infections [3–5], the investigation of a potential inhibition of the SARS-CoV-2 M^pro^ by direct factor Xa inhibitors, as suggested in the above virtual screening study, is of interest. In addition to evidence showing that apixaban decreases the mortality in COVID-19 positive patients [10,23], the aforementioned virtual screening study is not the only one reporting direct factor Xa inhibitors as potential inhibitors of the SARS-CoV-2 Mpro [24,25]. The fact that not all virtual screening studies identified the proposed factor Xa inhibitors can be due to several factors such as the screened data sets (e.g. natural compounds vs. selected protease inhibitors), the used receptor structures, the deployed molecular docking protocols, or the choice of pre- and post-docking methods and parameters. In this study, we therefore reassessed three factor Xa inhibitors—Apixaban, Betrixaban, Rivaroxaban, in a detailed computational protocol consisting of docking and molecular dynamics (MD) simulations to estimate the binding free energy of respective ligands. Notably, the predictions refer to non-covalent inhibitors as opposed to previously described covalent inhibitors [16,17].

Previous study showed that the binding cavity of SARS-CoV-2 M^pro^ features high flexibility and plasticity [26], thus potentially influencing the quality of the docking results as part of the virtual screening protocols. Those findings were also confirmed in other studies [27,28]. In order to test whether the selected compounds can bind to the SARS-CoV-2 M^pro^ and whether they can be considered as potential inhibitors of this molecular target, we performed binding affinity measurements with MicroScale Thermophoresis (MST) technique. Experimental studies allowed us to critically investigate the predictions made by the *in silico* methods on such challenging target as the SARS-CoV-2 M^pro^.

## Materials and methods

### Compounds preparation

Apixaban, Betrixaban and Rivaroxaban (Selleckchem) were dissolved in DMSO (according to standard formulations for *in vivo* experiments) to the final concentration of 10, 4 and 20 mM, respectively.

### Protein preparation

The concentration of the purified SARS-CoV-2 M^pro^ (Biosynth Carbosynth) was measured using the NanoDrop Spectrophotometer and BCA assay (Pierce™ BCA Protein Assay Kit, Thermo Fisher Scientific, Rockford, Illinois, USA) based on the beforehand prepared calibration curve on the bovine serum albumin.

The solution of the protein was diluted to the desired concentration with HEPES buffer (20 mM HEPES, 150 mM NaCl, 2 mM MgCl_2_, pH 7.0). In order to prevent aggregation and increase the stability of the protein, the solution of the protein was supplemented with Pluronic F-127 with the final concentration of 0.01% (w/v).

### Thermal stability analysis

#### The influence of DMSO concentration on the SARS-CoV-2 M^pro^ stability

The 3 μM unlabelled SARS-CoV-2 M^pro^ solution was tested with a series of concentrations of DMSO (from 0 to 5% v/v) after 2 hr incubation at room temperature. The experiment was performed on Standard Capillaries Prometheus NT.48 (NanoTemper, Munich, Germany) in two technical repetitions using the Prometheus NT.48 apparatus with the following parameters: excitation power: 100%, initial temperature: 20°C, final temperature: 80°C, and slope: 2°C/min.

#### Thermal stability of the SARS-CoV-2 M^pro^ with the examined compounds

Each solution of the examined compounds or DMSO was mixed with 4 μM SARS-CoV-2 M^pro^ solution in HEPES buffer with 0.01% (w/v) of Pluronic F-127 in separate samples. The concentrations were adjusted to limit the negative effect on protein stability. The final concentration of Apixaban was: 125 μM, Betrixaban: 50 μM and Rivaroxaban: 8.75 μM. The blank sample consisted of the SARS-CoV-2 M^pro^ with 1.25% DMSO. The experiment was performed on Standard Capillaries Prometheus NT.48 in two technical repetitions using the Prometheus NT.48 apparatus (NanoTemper, Munich, Germany) with the following parameters: excitation power: 100%, initial temperature: 20°C, final temperature: 70°C and slope: 2°C/min.

### The binding affinity of the compounds to the SARS-CoV-2 M^pro^

#### The intrinsic fluorescence of the compounds

The assessment of the fluorescence of the compounds was carried out based on the comparison of the fluorescence of the compound solutions in HEPES buffer with 0.01% (w/v) of Pluronic F-127 and SARS-CoV-2 M^pro^.

The concentrations of compounds were adjusted to limit the intrinsic fluorescence of the compounds. The final concentrations were: Apixaban: 125 μM, Betrixaban: 50 μM, Rivaroxaban: 140 μM, and SARS-CoV-2 M^pro^ solution: 4 μM.

The experiment was performed on LabelFree Capillary Chips in 2 technical repetitions using the Monolith NT.Automated (NanoTemper, Munich, Germany) with the following parameters: excitation power: 10% LabelFree, MST Power: medium, Before MST: 3s, MST-On Time 10s and After MST: 1s.

#### Binding affinity measurement—MST experiment

During the MST experiments, the concentration of the protein in solution was kept constant while the compounds were titrated. The dilution series of the compounds were prepared by applying a 3:1 ratio with initial concentrations of the compounds: Rivaroxaban: 17.5 μM, Apixaban: 250 μM, and Betrixaban: 100 μM. HEPES buffer supplemented with 0.01% of Pluronic F-127 was used to dilute the stock solutions of the compounds to the initial concentrations (mentioned above). The same buffer with 1.25% of DMSO (v/v) was used as the dilution buffer in the dilution series. Next, a constant amount of protein was added in 1:1 volume ratio to the respective diluted compounds resulting in final concentration of the protein of 4 μM and final concentration of the compounds starting from: Rivaroxaban: 8.75 μM, Apixaban: 125 μM and Betrixaban: 50 μM.

The experiments were performed on LabelFree Capillary Chips in 3 independent runs with one technical repetition using the Monolith NT.Automated (NanoTemper, Munich, Germany) with the following parameters: excitation power: 10% LabelFree, MST Power: medium, Before MST: 3s, MST-On Time 10s and After MST: 1s.

### *In silico* evaluation of the compounds

The Glide standard-precision (SP) [29] and smina [30] docking protocols were evaluated regarding their capability to reproduce crystallographic binding modes in an ensemble docking setting. An ensemble of eight structures was selected from 39 crystal structures (S1 Table) that contain 27 non-covalent co-crystallized ligands. The performance of the docking protocols were determined using a root mean-square deviation (RMSD) threshold of 2.5 Å with respect to the native binding mode. The best-performing ensemble in the Glide SP protocol was retained for further procedures (S2 Table). For these procedures, the protein structures were pre-processed with the Protein Preparation Wizard [31] implemented in the Schrodinger Small-Molecule Drug Discovery Suite [32] with default specifications except for a pH value of 7.4 for calculations involving ionization. For the following production phase, we preprocessed the three ligand structures with the LigPrep [33] routine in Maestro to obtain energy-minimized 3D conformers with the OPLS3e force field. The protonation states were predicted at pH 7.4 with Epik. The ligands were docked to the selected ensemble and the complexes with the lowest score for each compound were retained. Next, MD simulations were conducted with the Desmond (v2019-1) [34] engine using the OPLS_2005 force field in an NpT ensemble. The temperature was controlled by the Nose-Hoover thermostat and atmospheric pressure was maintained by the Martyna-Tobias-Klein barostat. By default, long-range forces were treated with the u-series algorithm [35] and a cutoff of 9 Å for short-range interactions. We constrained bonds containing hydrogen atoms with the M-SHAKE algorithm, while the orthorhombic periodic systems were solvated with TIP3P water molecules and an appropriate number of counter ions zeroing the net charge of the simulated system. After the default relaxation protocol, every complex was simulated in triplicates for 50 ns at 310 K. The time step of the RESPA integrator was set to 2 fs and atomic coordinates were saved at an interval of 10 ps. The obtained trajectories were then subjected to the Molecular Mechanics Generalized Born Surface Area (MM/GBSA) protocol using the thermal_mmgbsa.py script in Maestro to obtain binding free energies for each ligand. Every second frame of the last 10 ns was selected to be processed by the routine and the average interaction energies were computed.

## Results

### Thermal stability of the SARS-CoV-2 M^pro^ with the examined compounds

Prior to binding affinity experiments, we used the nanoDSF method (Prometheus NT.48, NanoTemper) to test the stability of the SARS-CoV-2 M^pro^. We determined the effect on the protein stability at different concentrations of DMSO (ranging from 0 to 5% v/v) and in the presence of the tested compounds. The results indicate that the amount of DMSO in solution should be not higher than 1% in order not to affect the protein stability. Higher concentration of DMSO (5% v/v) in solution lowers the melting temperature (T_m_) value from 55.8^०^C to 54.8^०^C (S1 Fig, S3 Table). The thermal stability of the SARS-CoV-2 M^pro^ is not affected by the finally settled concentrations of all compounds (S2 Fig, S4 Table).

### The binding affinity of the compounds to the SARS-CoV-2 M^pro^

To avoid any potential conformational changes of the protein that could be caused by protein labelling, we measured the binding affinity of the compounds to the SARS-CoV-2 M^pro^ during the label-free MST experiment on Monolith NT.Automated (NanoTemper). We measured the native fluorescence of the compounds and compared it to the fluorescence of the protein. All compounds were diluted to the highest concentrations (limited by their solubility) in HEPES buffer. The results indicate that the fluorescence of Rivaroxaban, Apixaban and Betrixaban does not exceed the fluorescence threshold in the label-free binding affinity experiments at the concentrations 140 μM, 125 μM and 50 μM, respectively (S3 Fig).

We measured the binding affinity of all compounds using 4 μM SARS-CoV-2 M^pro^. The protein was titrated with the compounds with previously determined highest concentration of the compounds that could be used due to the native fluorescence restriction.

**Fig 1:**
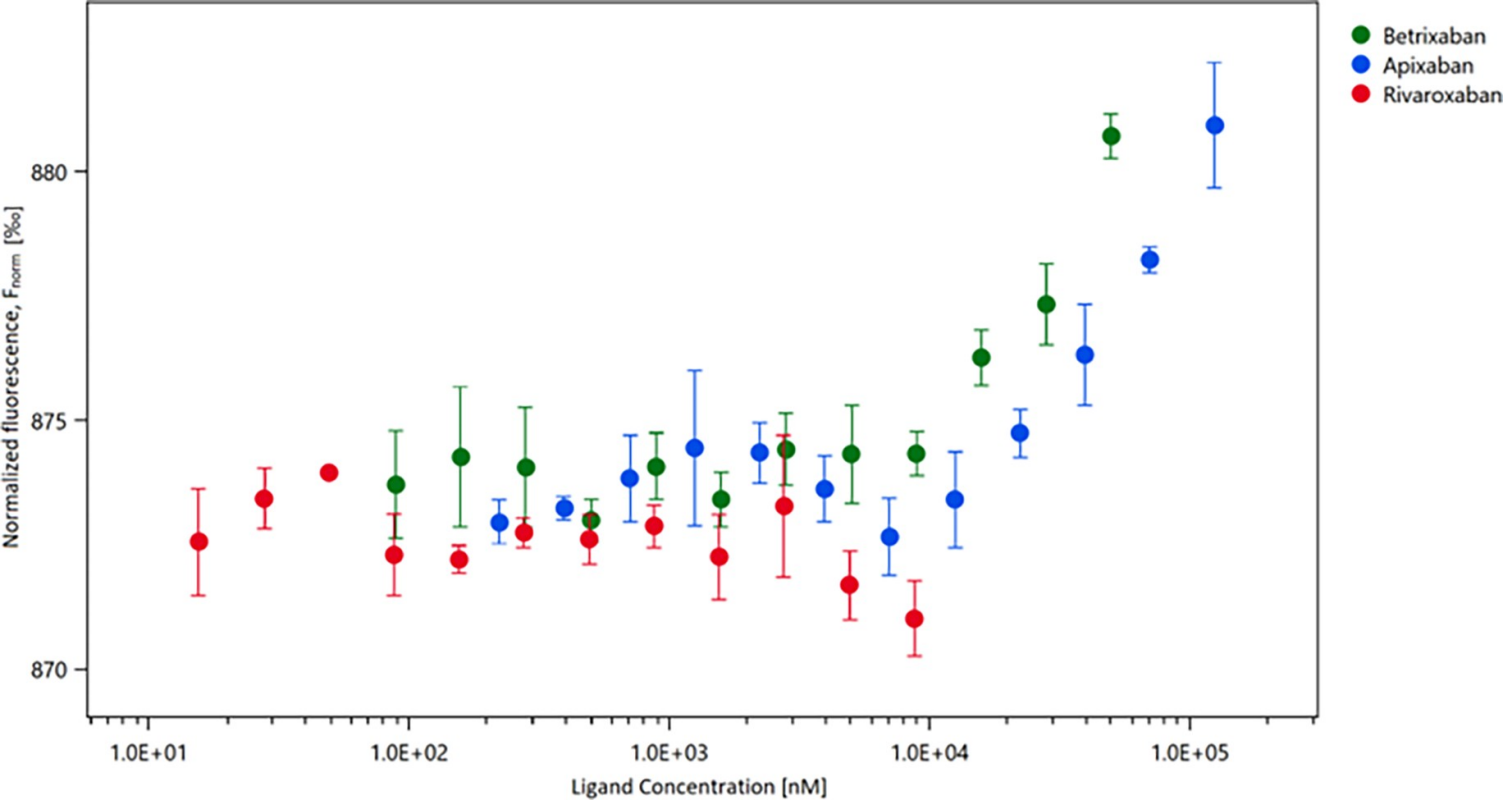
Results of the change in normalized fluorescence depending on the concentration of the ligand.

The experiment shows that in the tested concentration range, interaction between Rivaroxaban and SARS-CoV-2 M^Pro^ is not observed (Fig 1). In the case of the Apixaban and Betrixaban, a signal for the binding to SARS-CoV-2 M^Pro^ can be observed. In the high range of the measured concentrations of compounds, the signal from fluorescence is increasing, however a saturation of the signal is not achieved. Therefore, the exact K_d_ value of the Apixaban and Betrixaban cannot be calculated (Fig 1). A higher concentration of the compounds could be achieved by an increase in DMSO concentration, however, due to negative effects on the SARS-CoV-2 M^Pro^ stability we did not apply such a procedure.

### *In silico* evaluation of the compounds

Our assessment of the smina and Glide docking protocols regarding their capability to reproduce crystallographic binding modes revealed the latter to be more reliable. Using Glide in an ensemble docking setting, the binding modes of 26 ligands (96.3%) could be correctly reproduced within a RMSD threshold of 2.5 Å (S2 Table). The best-scored complexes from docking were subjected to MD simulations followed by MM/GBSA post-processing. The obtained binding free energies are presented in S5 Table. Even though both docking and the MM/GBSA protocol predicted the best score for Apixaban, the results regarding Betrixaban and Rivaroxaban were inconclusive between the different scoring procedures.

## Discussion

Anticoagulant treatment with factor Xa inhibitors has been shown to have positive effects on severely ill patients infected with SARS-CoV-2 due to the reduction of thromboembolic events. As previous *in silico* studies suggested that Apixaban, Rivaroxaban and Betrixaban could be potential inhibitors of SARS-CoV-2 M^pro^, we investigated a possible dual mechanism of these direct factor Xa inhibitors. Here we presented our efforts for experimental determination of the binding affinity of those three compounds using the MST technique. Our results indicate that there is no binding of the Rivaroxaban to SARS-CoV-2 M^pro^, and the binding of the Apixaban and Betrixaban can only be observed at rather high concentrations of the compounds. Unfortunately, the K_d_ values of examined compounds could be potentially measured only with the use of high concentrations of the compounds that, unfortunately, cannot be reached due to the low solubility of the compounds in buffer and distortion of the protein stability in higher DMSO content (higher than 2.5%). It should be emphasised that higher concentrations of the compounds are also not achievable in blood plasma during standard treatment conditions [36,37]. Even though the binding free energies obtained from the combination of MD simulations and MM/GBSA calculations offered promising results when we compared them to several co-crystallized inhibitors of M^pro^ reported in our previous work [18], the experiments performed in this study did not confirm the expectations. Moreover, recent findings suggest a high risk of the strong side effects of the drugs targeting the active site of SARS-CoV-2 M^pro^ due to the high similarity of the active site cavity with those of cysteine and serine proteases [38]. All these results confirm earlier suggestions [26] that SARS-CoV-2 M^pro^ can be a difficult target for basic screening approaches due to the high flexibility and plasticity of the active site pocket and underlines risk of the overestimation of binding energies of large molecules. They reflect also the fact that docking scores and results of MM/GBSA calculations scales with molecular weight and therefore the binding scores of large molecules can be overestimated in comparison to smaller molecules.

## Supporting information

S1 Fig The comparison of the stability of SARS-CoV-2 M^pro^ in buffer and with different DMSO concentration. (TIF)

S2 Fig The analysis of the influence of the Apixaban, Rivaroxaban and Betrixaban on the thermostability of the SARS-CoV-2 M^pro^. (TIF)

S3 Fig The comparison of the native fluorescence of the Apixaban, Rivaroxaban and Betrixaban with the fluorescence of the protein. (A) Capillary position 1,2: 4 μM unlabeled protein + 1.25% DMSO; Capillary position 3,4: 125 μM Apixaban. (B) Capillary position 1,2: 4 μM unlabeled protein + 1.25% DMSO; Capillary position 3,4: 250 μM Rivaroxaban; Capillary position 5,6: 187.5 μM Rivaroxaban; Capillary position 7,8: 140 μM Rivaroxaban; Capillary position 9, 10: 50 μM Betrixaban.
(TIF)

S1 Table Crystal structures examined in cross-docking. Selected structures for the docking production runs are marked by an asterisk.
(DOCX)

S2 Table Results from ensemble docking. The percentage indicates how many binding modes could be reproduced with an RMSD below 2.5 Å.
(DOCX)

S3 Table The measured melting temperatures of SARS-CoV-2 M^pro^ in a buffer, with different DMSO concentration. (DOCX)

S4 Table The measured melting temperatures of SARS-CoV-2 M^pro^ in a buffer with all tested compounds. (DOCX)

S5 Table Results of the *in silico* evaluation of the analysed compounds. (DOCX)
