# Supplementary figures and images for: Evaluation of Xa inhibitors as potential inhibitors of the SARS-CoV-2 Mpro protease
Source: PLoS One. 2022 Jan 11;17(1):e0262482. doi: 10.1371/journal.pone.0262482 (PMC8752003; doi:10.1371/journal.pone.0262482)

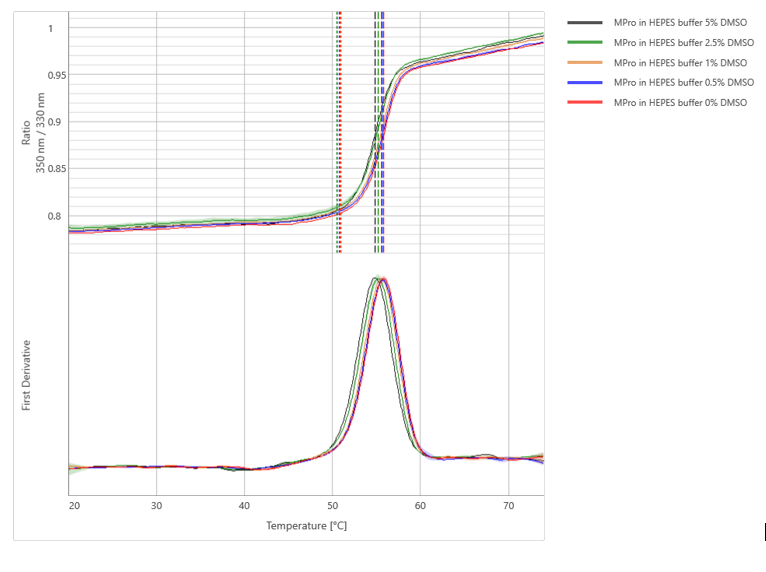

Supplement: S1 Fig — (TIF) [file pone.0262482.s001.tif]

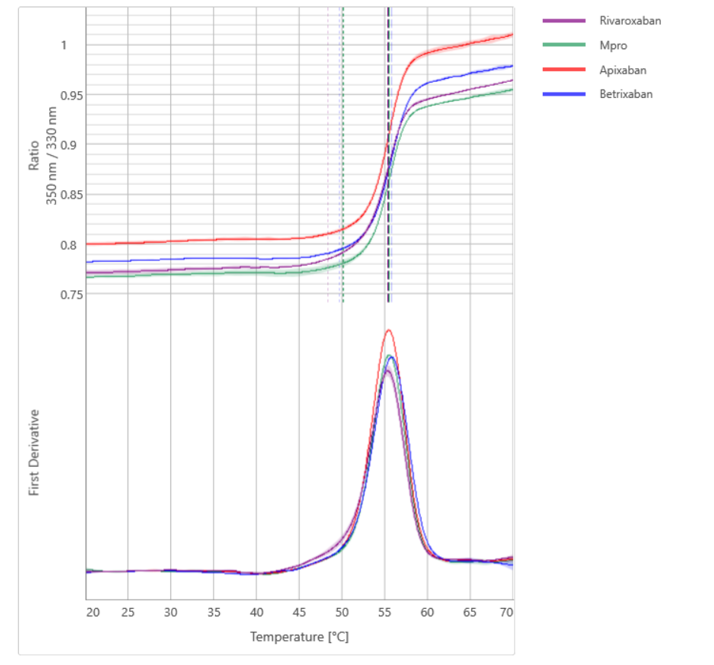

Supplement: S2 Fig — (TIF) [file pone.0262482.s002.tif]

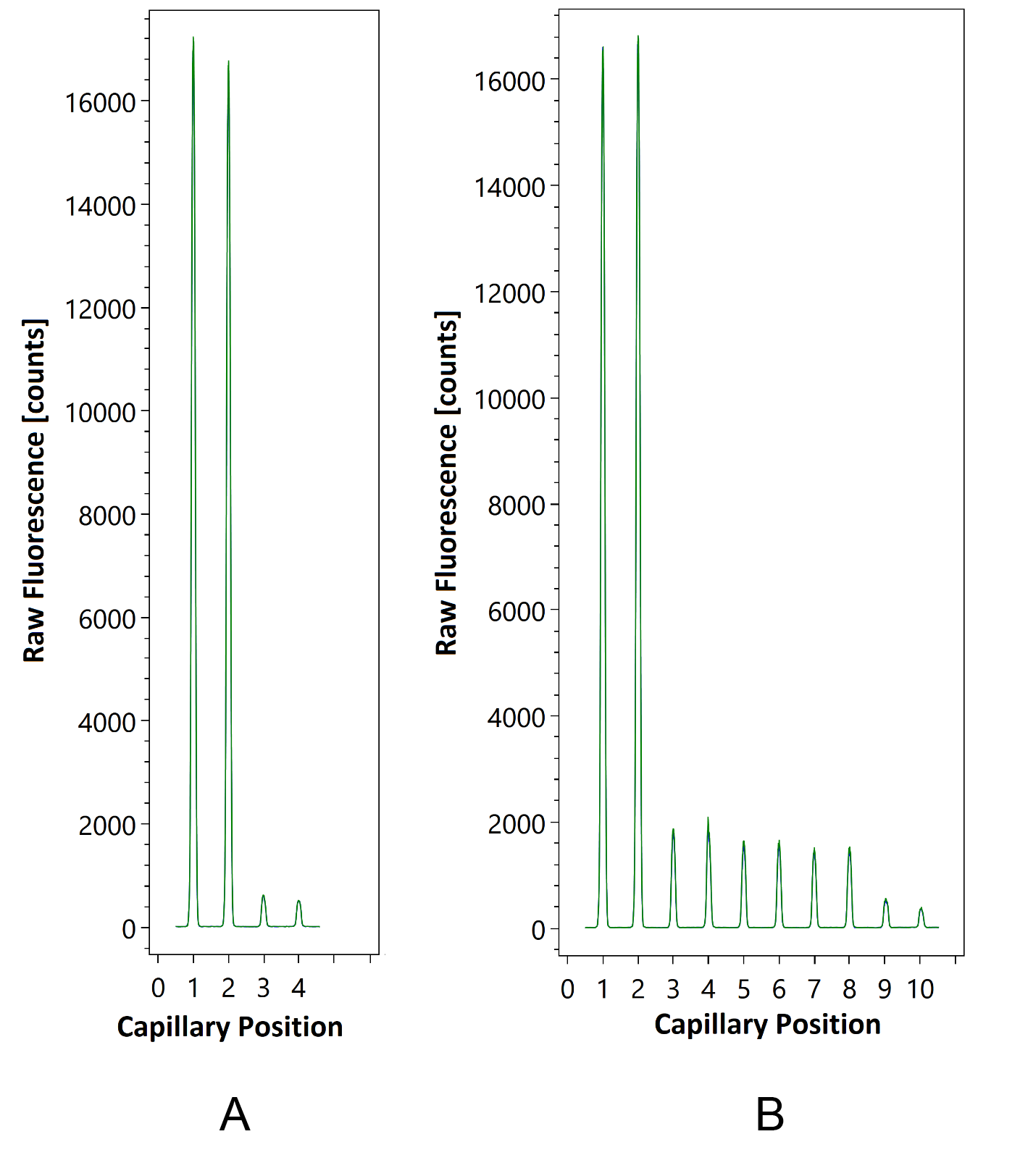

Supplement: S3 Fig — (A) Capillary position 1,2: 4 μM unlabeled protein + 1.25% DMSO; Capillary position 3,4: 125 μM Apixaban. (B) Capillary position 1,2: 4 μM unlabeled protein + 1.25% DMSO; Capillary position 3,4: 250 μM Rivaroxaban; Capillary position 5,6: 187.5 μM Rivaroxaban; Capillary position 7,8: 140 μM Rivaroxaban; Capillary position 9, 10: 50 μM Betrixaban. (TIF) [file pone.0262482.s003.tif]
