# Supplementary material for: Evaluation of Xa inhibitors as potential inhibitors of the SARS-CoV-2 Mpro protease
Source: PLoS One. 2022 Jan 11;17(1):e0262482. doi: 10.1371/journal.pone.0262482 (PMC8752003; doi:10.1371/journal.pone.0262482)
Supplement: S1 Table — Selected structures for the docking production runs are marked by an asterisk. (DOCX) [file pone.0262482.s004.docx]

| PDB IDs |
| --- |
| 5R7Y, 5R7Z, 5R8T*, 5R80, 5R81*, 5R82, 5R83, 5R84, 5RE4, 5REB, 5REZ*, 5RF1,5RF3, 5RF, 5RF7 5RFE, 5RG1, 5RGH, 5RGI, 5RGU, 5RGV, 5RGW, 5RGX, 5RGY,5RGZ, 5RH0, 5RH1, 5RH2, 5RH3*, 5RH8, 6LU7*, 6LZE, 6M0K, 6M2N, 6M2Q, 6W63, 6Y2E, 6YB7, 7BQY, 7BRO, 7BUY* |
