# Supplementary material for: Evaluation of Xa inhibitors as potential inhibitors of the SARS-CoV-2 Mpro protease
Source: PLoS One. 2022 Jan 11;17(1):e0262482. doi: 10.1371/journal.pone.0262482 (PMC8752003; doi:10.1371/journal.pone.0262482)
Supplement: S2 Table — The percentage indicates how many binding modes could be reproduced with an RMSD below 2.5 Å. (DOCX) [file pone.0262482.s005.docx]

| **Ensemble size** | **Percentage** | **Protocol** |
| --- | --- | --- |
| 4 | 81.5% | Glide SP |
|  | 63.0% | smina |
| 5 | 85.2% | Glide SP |
|  | 66.7% | smina |
| 6 | 88.9% | Glide SP |
|  | 70.4% | smina |
| 7 | 92.6% | Glide SP |
|  | 70.4% | smina |
| 8 | 96.3% | Glide SP |
