# Supplementary material for: Evaluation of Xa inhibitors as potential inhibitors of the SARS-CoV-2 Mpro protease
Source: PLoS One. 2022 Jan 11;17(1):e0262482. doi: 10.1371/journal.pone.0262482 (PMC8752003; doi:10.1371/journal.pone.0262482)
Supplement: S3 Table — (DOCX) [file pone.0262482.s006.docx]

| **Analysed system** | **Additives** | **T_m_ [°C]** | **ΔT_m_ [°C]** |
| --- | --- | --- | --- |
| SARS-CoV-2 M^pro^ | 0% DMSO | 55.80°C | ±0.02°C |
|  | 0.5% DMSO | 55.69°C | ±0.05°C |
|  | 1% DMSO | 55.53°C | ±0.08°C |
|  | 2.5% DMSO | 55.16°C | ±0.04°C |
|  | 5% DMSO | 54.83°C | ±0.01°C |
