# Supplementary material for: Evaluation of Xa inhibitors as potential inhibitors of the SARS-CoV-2 Mpro protease
Source: PLoS One. 2022 Jan 11;17(1):e0262482. doi: 10.1371/journal.pone.0262482 (PMC8752003; doi:10.1371/journal.pone.0262482)
Supplement: S4 Table — (DOCX) [file pone.0262482.s007.docx]

| **Analysed system** | **Buffer** | **Additives** | **T_m_ [°C]** | **ΔT_m_ [°C]** |
| --- | --- | --- | --- | --- |
| SARS-CoV-2 M^pro^ | HEPES | 1.25 % DMSO | 55.50°C | ±0.03°C |
|  |  | 125 μM of **Apixaban** | 55.43°C | ±0.01°C |
|  |  | 50 μM of **Betrixaban** | 55.75°C | ±0.05°C |
|  |  | 8.75 μM of **Rivaroxaban** | 55.31°C | ±0.08°C |
