# Supplementary material for: Evaluation of Xa inhibitors as potential inhibitors of the SARS-CoV-2 Mpro protease
Source: PLoS One. 2022 Jan 11;17(1):e0262482. doi: 10.1371/journal.pone.0262482 (PMC8752003; doi:10.1371/journal.pone.0262482)
Supplement: S5 Table — (DOCX) [file pone.0262482.s008.docx]

| **Ligand** | **Glide score (kcal/mol)** | **∆G_MM/GBSA_ (kcal/mol)** |
| --- | --- | --- |
| Apixaban | -7.8 | -77.9 ± 9.3 |
| Betrixaban | -7.0 | -57.3 ± 2.3 |
| Rivaroxaban | -6.9 | -73.9 ± 2.5 |
